# A review of Hallermann-Streiff syndrome demonstrates clinical overlap with other conditions

**DOI:** 10.1186/s13023-026-04277-7

**Published:** 2026-02-25

**Authors:** Caroline Cavender, Carrie Atzinger, Anne Slavotinek

**Affiliations:** 1 Division of Human Genetics, Cincinnati Children’s Hospital Medical Center, Department of Pediatrics, Cincinnati, OH USA; 2 University of Cincinnati College of Medicine, Cincinnati, OH USA

**Keywords:** Hallermann-Streiff syndrome

## Abstract

Hallermann-Streiff syndrome (HSS) is a rare genetic condition characterized by seven cardinal findings comprising congenital cataracts, microphthalmia, recognizable facial features, sparse hair with hypotrichosis, skin atrophy, dental anomalies, and short stature. Around 200 patients have been reported and almost all cases have been sporadic. Several reviews have described the clinical findings in patients reported to have HSS, but the lack of a genetic test or other biomarker has complicated an accurate delineation of the condition. Although HSS is suspected to have a genetic basis and non-recurrent deleterious variants in genes such as *GJA1*, *CHD6*, and *ZMPSTE24* have been noted in patients diagnosed with HSS, the etiology for almost all cases remains unknown. Our findings reveal a consistent phenotype but highlight the diagnostic challenges associated with HSS, including clinical overlap with other genetic conditions. It is also likely that the original reports of HSS described patients with heterogeneous conditions. Further research to identify the cause(s) of HSS and to develop best practices for patient care are needed.

**Supplementary Information:**

The online version contains supplementary material available at https://doi.org/10.1186/s13023-026-04277-7.

## Introduction

Hallermann-Streiff syndrome (HSS, OMIM 234100; also known as oculo-mandibulodyscephaly with hypotrichosis syndrome), is a rare condition with an unknown etiology [1, 2]. HSS is rare, with around 200 patients reported from around the world [2]. The incidence was estimated in a 1990 survey that identified 1/27,472 newborns with HSS in Japan [3, 4]. The condition takes its name from reports describing the characteristic facial appearance and congenital cataracts [5] and mandibulofacial malformation and ocular changes [6], although a patient with possible HSS was first described in 1893 [7]. HSS was later defined as a syndrome with seven characteristic findings: (i) a recognizable facial gestalt (previously called a ‘bird-like’ face), (ii) dental anomalies, (iii) proportionate short stature (previously called ‘proportionate nanism’), (iv) hypotrichosis, (v) atrophy of the skin, (vi) microphthalmos, and (vii) congenital cataracts [8]. The cardinal facial features have been the most consistent finding throughout the years and have been considered mandatory for the diagnosis [2]. Most patients diagnosed with HSS have been sporadic, without affected parents or siblings, and the inheritance pattern remains unknown [2, 9].

The phenotype associated with HSS was recently reviewed in 21 patients [10]. The review found that craniofacial features were present in all patients, with a ’beaked’ nose in 100%, micrognathia/retrognathia in 96%, and frontal or parietal bossing in 89% [10]. In the same review, bilateral microphthalmia was present in 81%, bilateral congenital cataracts in 69%, and proportionate short stature in 69% of patients [10]. Hypotrichosis, alopecia, dental anomalies, and atrophy of the skin were also found in more than two-thirds of patients [10]. Pertinent negative findings suggestive of an alternative diagnosis were identified in less than a third of the 21 patients and included ear anomalies and abnormal palpebral fissures (indicative of mandibulofacial dysostosis), muscular atrophy, chronic arthritis and premature arteriosclerosis (indicative of progeroid conditions), limb anomalies (indicative of oculodentodigital dysplasia; ODDD; OMIM 164200), and significant intellectual disability [8].

The clinical definition of HSS has been challenging due to relatively low patient numbers, lack of a clearly defined inheritance pattern and causative gene(s), and absence of a diagnostic biomarker. We reviewed 24 patients reported to have HSS from 43 published papers reported over the last 20 years to describe the phenotypic findings associated with this condition. Our review differs from recent past reviews [2, 10] in its focus on overlapping genetic conditions and in proposing management guidelines.

## Materials and methods

### Literature review

We reviewed 43 articles featuring patients diagnosed with HSS that had been published in the medical literature since 2003. The search criteria included papers with “Hallermann-Streiff Syndrome” in the title. After excluding cases with sparse clinical details, reviews without new patient information, and papers that were not written in English, we compiled clinical findings and genetic testing results for 24 patients (Supplementary Table S1; [11] (7 patients); [12, 13] (2 patients); [14] (2 patients); [15, 16] (4 patients); [17–23]).

## Results

**Fig. 1:**
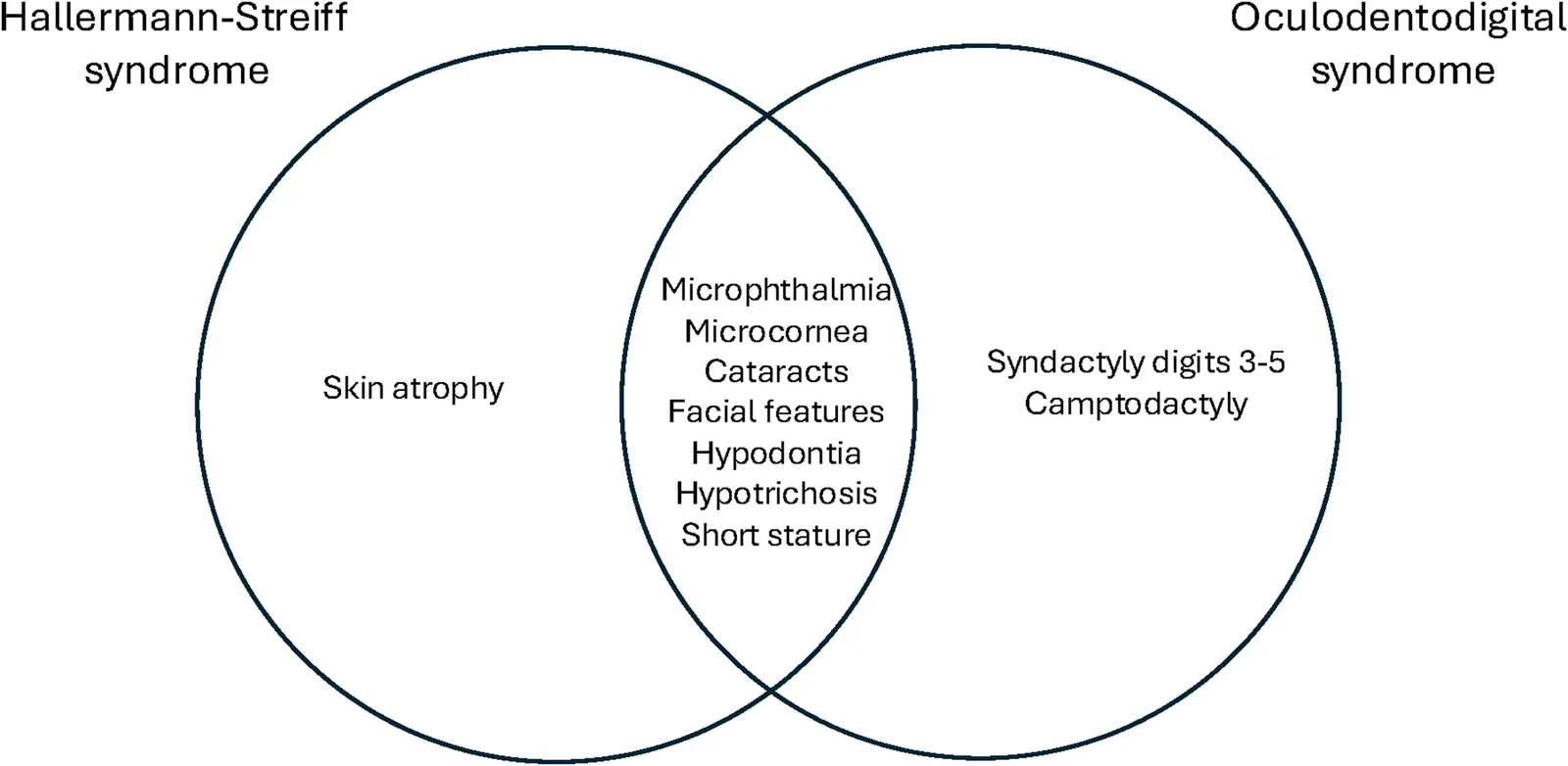
Diagram showing clinical overlap between Hallermann-Streiff syndrome and oculodentodigital dysplasia. Features seen in each disorder are included in the relevant circle, showing overlap for microphthalmia, cataracts, microcornea, hypodontia, hypotrichosis and short stature

Patients with phenotypic overlap with other conditions were excluded from the final group of 24 patients. The most frequent clinical overlap was with ODDD syndrome (Fig. 1), which shares eye findings of microphthalmia and microcornea, facial features, and hypodontia, but is distinguishable from HSS due to camptodactyly and syndactyly involving the 3rd -5th digits ([7, 24–26]). In our literature review, we identified three patients with syndactyly that may have had ODDD [27–29], although genetic testing for *GJA1* variants was not performed. The first patient was a five-year old female born to consanguineous parents who had congenital cataracts, small orbits, frontal bossing, a beak-shaped, tapering nose, midface hypoplasia and retrognathia, sparse hair, eyelashes and eyebrows, atrophy of the facial skin, proportionate short stature, and syndactyly of the fingers [27]. The digits affected by the syndactyly were not specified [27]. The second patient was a three-year old female with microphthalmia, blue sclerae and strabismus, classical facial features, sparse hair and eyebrows, skin atrophy, hypoplasia of the primary and secondary dentition, and short stature [28]. This child had bilateral syndactyly of the 4th and 5th fingers [28]. A 23-year-old female was reported to have HSS, but also had syndactyly of the 4th and 5th fingers, making ODDD likely [29]. She presented with multiple symptoms, including generalized pain in her teeth and a history of recurrent respiratory infections. Upon examination, she exhibited several characteristic features of HSS, such as a brachycephalic head, a ‘parrot-beak’ nose, rough-textured thin hair, microphthalmia, hypertelorism, and strabismus, but had complete fusion of the 4th and 5th digits of her hands [29]. These three cases are good examples of the strong clinical overlap between these two conditions.

We also excluded several patients in whom congenital cataracts and colobomas appeared to be part of a complex eye phenotype. A pair of monozygotic female twins were reported to have all seven features of HSS including congenital cataracts [30, 31]. However, both girls had exudative retinal detachments, and one had a corneal descemetocele that has not previously been observed in HSS [30, 31]. We did include the patients of AlAli et al. [18] and Carreras-Castañer et al. [20], who also had additional ocular findings. Other patients reported to have the characteristic facial gestalt of HSS have also had significantly reduced growth that may be more consistent with another diagnosis. For example, one adult patient had a height of 143 cm (< 3rd centile; 50th centile for age 10–11 years) and weight of 38 kg (< 3rd centile; 50th centile for age 11–12 years; [32]). A third patient had a height of 94 cm and a weight of 12 kg at five years and four months of age and thus had severe growth delays [33]. We did not find patients with an age of onset after one year who had a phenotype resembling progeria.

The frequency of the cardinal findings in the 24 patients with HSS are summarized in Tables 1 and 2 and are provided in full in Supplementary Table S1. There were 10 males and 14 females, and the age of diagnosis ranged from infancy to adulthood. None of the patients included in our study had a family history of the condition. Genetic testing in these patients was very limited. Three of the patients had a normal karyotype, and only two patients had exome sequencing which was reported as non-diagnostic [20, 21]. Although one patient was delivered prematurely, there were no other complications reported in pregnancy. Growth parameters at birth were in the typical range except for one child born prematurely who had intrauterine growth restriction for length and weight [20], one infant with a length <5th centile, one infant with a head circumference <5th centile [11] and one baby with reduced weight <5th centile [17].

**Table 1:** Frequency of seven cardinal findings in Hallermann-Streiff syndrome

Feature	Schmidt and Wollnik, [2]	Preudhomme et al., 2022	This study
Facial gestalt^1^	Constant	100%	24/24 (100%)
Congenital cataracts	Common	69%	20/24 (83%)
Microphthalmia	Common	81%	18/24 (75%)
Hypotrichosis	Common	88%	17/24 (71%)
Abnormal dentition	Common	85%	17/24 (71%)
Short stature	Common	69%	14//24 (58%)
Atrophy of skin	Common	73%	12/24 (50%)

Classical facial features or a smaller nose with retro-micrognathia was required

**Table 2:** Clinical findings for 24 patients diagnosed with Hallermann-Streiff syndrome

*Clinical finding*	*Numbers*
***Facial features***	
Classical facial appearance	Yes: 11/24; No or not noted 13/24; 22/24 small/short/thin nose and micro-retrognathia
Brachycephaly	Yes: 2/24, No or not noted: 22/24
Frontal bossing/broad forehead	Yes: 9/24, No or not noted: 15/24
Wide-set eyes	Yes: 0/24, No or not noted: 24/24
Midface hypoplasia	Yes: 1/24, No or not noted: 23/24
Narrow nasal bridge	Yes: 4/24; No or not noted: 20/24
Small nose/nares	Yes: 18/24; No or not noted: 6/24
Hypoplastic nasal cartilage	Yes: 2/24, No or not noted: 22/24
Microstomia	Yes: 7/24; No or not noted: 17/24
Micrognathia	Yes: 15/24, No or not noted: 9/24
Retrognathia	Yes: 12/24, no or not noted: 12/24
Large tongue	Yes: 1/24, No or not noted: 23/24
High-arched palate	Yes: 5/24, No or not noted: 19/24
***Ocular***	
Cataracts	Yes: 20/24, No or not stated: 4/24; congenital in 18/20
Corneal opacity	Yes: 1/24; No or not noted: 23/24
Microphthalmia	Yes: 18/24; No or not noted: 6/24
Abnormal retinae	Yes: 5/24, No or not noted: 19/24
Blue sclerae	Yes: 3/25, No or not noted: 21/24
Microcornea	Yes: 6/24, No or not noted: 18/24
Nystagmus	Yes: 5/24, No or not noted: 19/24
Strabismus	Yes: 7/24, No or not noted: 17/24
***Dental findings***	
Natal teeth	Yes: 7/24, No or not noted: 17/24
Dental hypoplasia	Yes: 2/24, No or not noted: 22/24
***Ear***,*** Nose and Throat (ENT)***	
Choanal atresia/hypoplasia	Yes: 1/24; No or not noted: 23/24
Snoring	Yes: 2/24, No or not noted: 22/24
Sleep apnea	Yes: 3/24, No or not noted: 21/24
***Dermatology***	
Thin or sparse hair/hypotrichosis	Yes: 17/24, No or not noted: 7/24
Alopecia	Yes: 1/24, No or not noted: 23/24
Skin atrophy/thinning	Yes: 12/24, No or not noted: 12/24
Telangiectasias	Yes: 1/24, No or not noted: 23/24
***Growth***	
Microcephaly	Yes: 3/24, No or not stated: 21/24
Failure to thrive	Yes: 3/24, No or note stated: 21/24
Short stature	Yes: 14/24; No or not stated 10/24

All 24 patients had eye findings which included cataracts (20/24; 83%) that were congenital in 18/20 (90%) and/or microphthalmia (18/24; 75%). Microcornea was present in 6/24 (25%). However, the eye findings in these reports were variable and included strabismus (7/24; 29%), nystagmus (5/24; 21%), abnormal retinae (5/24; 21%), blue sclerae (3/24; 13%) and corneal opacity (1/24; 3%). Three patients had isolated posterior synechiae [16]. Glaucoma or raised intraocular pressure needing surgery was present in two patients and papilledema in one patient [11, 13]. There were single reports of chronic anterior uveitis [13], a small optic nerve head [13], corneal edema and Brown-McLean syndrome [15], iris atrophy with diffuse posterior synechia in both eyes with coloboma of optic nerve [16], bilateral iris and fundal colobomas [17], enophthalmos [20], and a blurred optic disk margin, pallor around the macular area, and widespread pigmented spots in the peripheral retina [18].

All patients were reported to have either a ‘classical’ facial appearance or ‘beak-like’ nose, or had a combination of a small, short and/or thin nose with micro-retrognathia. Hypotrichosis (17/24; 71%) and skin atrophy (12/24; 50%) were also frequent. 17/24 (71%) patients had dental anomalies, including 7/24 (29%) individuals with natal teeth. Facial features comprised small nose/nares in 18/24 (75%), micrognathia in 15/24 (63%), retrognathia in 12/24 (50%), frontal bossing/broad forehead in 9/24 (38%) and microstomia in 7/24 (29%). A high anterior hairline was noted in 7/24 (29%) of patients. 5/24 (21%) had a high-arched and/or narrow palate and 3/24 had mandibular hypoplasia (13%). Rarer findings were a narrow nasal bridge (4/24; 17%), brachycephaly (2/24; 8%), downslanting palpebral fissures (2/24; 8%), short neck (2/24; 8%), and hypoplastic nasal cartilages (2/24; 8%). There were single instances of midface hypoplasia and a large tongue. Three patients had minor ear anomalies (13%).

In addition to thin or sparse hair/hypotrichosis (17/24; 71%) and skin atrophy or thin skin (12/24; 50%), one patient had alopecia of the scalp [14] and one patient had skin telangiectasias [11]. Dental findings included natal teeth in 7/24 (29%) and dental hypoplasia or hypodontia in 2/24 (8%). There was a broad range of other dental anomalies, including dental crowding and abnormally formed teeth. Malocclusion was present in 3/24 (13%). Postnatal growth was variable, and heights ranged from a Z score of -4.4 to -1.5. 14/24 (58%) had short stature as defined by a height <5th centile or ‘growth delay’. Postnatal weight was reduced in 5/24 (21%).

HSS is not typically associated with developmental delays, but 8/24 (33%) patients had neurocognitive delays which were reported to improve in two out of the eight patients. Three patients had motor delays and walked later than 16 months of age, but the motor delays in one of these patients also resolved [11]. Two patients (2/24; 8%) had hypotonia [18, 21] and none were reported to have cerebral malformations, seizures, cerebral palsy or developmental regression. One patient had two small ventricular septal defects and an atrial septal defect [21] and another had a small patent ductus arteriosus that was repaired at six months [17]. One patient had a submucous cleft of the palate [17] and one had a micropenis [14] but renal abnormalities were not noted. One patient had narrowing of the choanae [21].

Regarding medical complications of HSS, 7/24 (29%) patients were noted to have recurrent respiratory tract infections, pneumonias, or airway disease. Recurrent infections of the upper airways and apnea, recurrent pneumonias and apnea [11], recurrent chest infections [17], ‘airway problems’ [22], and chronic atelectasis with recurrent pulmonary infections [18] were noted. One patient had respiratory distress after birth [11]. Snoring [11, 12], obstructive sleep apnea (OSA: [18, 21]) and OSA requiring mechanical ventilation [14] were also present. Other management concerns included a need for care with anesthesia [34] and many reports highlighted a need for specialist dental care and interventions for a small jaw. Three patients had feeding difficulties. Two patients had gastroesophageal reflux disease (GERD; [14, 21]), with one requiring a gastrostomy and Nissan fundoplication [14]. Single patients had aspiration [21] and velopharyngeal insufficiency [11].

Lastly, we noted several patients who had a similar constellation of skeletal findings in additional to the characteristic features ascribed to HSS ([11, 35–37]). These patients had blue sclerae, natal teeth and brittle and irregular teeth, thin ribs, clavicles and metacarpals, thin and gracile tubular bones, and small vertebrae ([35–37]). In particular, the ulna and radius were thin, and two patients had a fracture of the radius that was described as spontaneous in one patient ([36, 37]). Other skeletal findings in these patients and others described to have HSS include hip dysplasia [11], asymmetry of the lower limbs ([11, 17]) and facial asymmetry [19]. Poorly ossified and thin calvaria, large fontanelles with wide separation of the cranial sutures, winging of the scapulae, cervical-thoracic scoliosis, clubfoot, plano-valgus deformity and an impression of short arms and legs in comparison to body length ([11, 35–38]). Skin findings were also more prominent, with redundant dry and wrinkled skin and ‘leathery’ skin with prominent vessels [11, 35]. Members of this group with additional skeletal findings also had severe short stature with a low IGF1 level and multiple anomalies affecting other body systems, including hypothyroidism, congenital heart disease, polymicrogyria with hypoplasia of the corpus callosum, cerebellum and basal ganglia, and unilateral choanal atresia [11, 35]. Lastly, fractures were also noted in a male with bilateral cataracts who died in the neonatal period and had a female sibling who was delivered at 29 weeks of gestation with identical facial features resembling a severe form of HSS [38]. Both siblings had slender long bones in addition to the fractures and the skull was under-ossified [38]. The recurrence in a male and female sib pair was suggestive of autosomal recessive inheritance or a dominant mutation with parental germline mosaicism [38]. It is unclear if these patients have HSS or a different, overlapping condition.

## Discussion

**Fig. 2:**
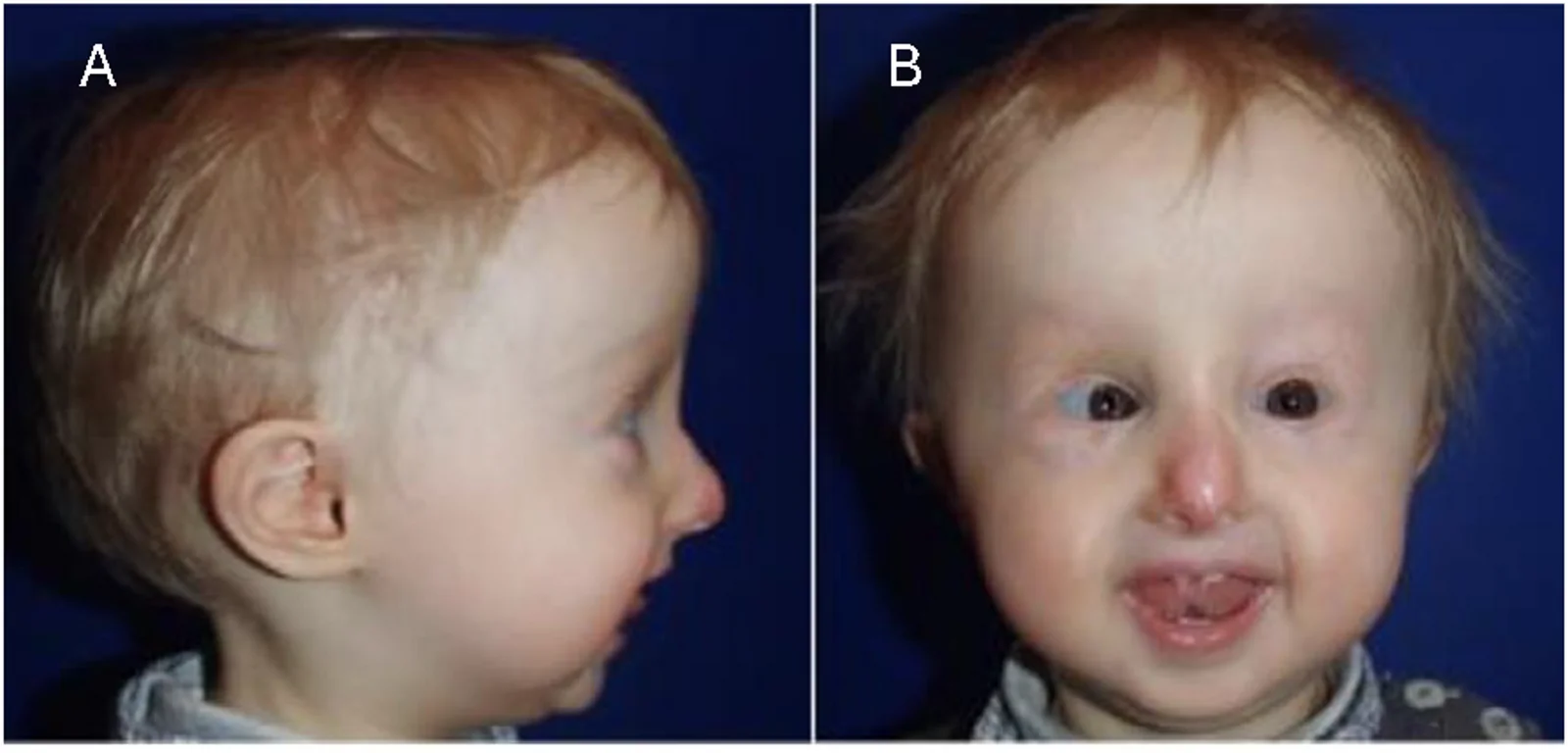
Facial and profile photograph of a child with Hallermann-Streiff syndrome, showing facial anomalies with a relatively large-appearing cranium, sparse hair, eyebrows and eyelashes, a prominent forehead with a high anterior hairline, deep-set eyes with relatively small palpebral fissures, blue sclerae and strabismus, a thin nasal bridge and tip with small alae nasi, and a small and retruded jaw. Reproduced with permission from Preudhomme et al. “Hallermann Streiff syndrome: Cranio-facial manifestations systematic review and report of two cases”, Journal of Stomatology oral and Maxillofacial Surgery, 2022;123:e219-e223

HSS was first identified as an independent syndrome in a paper that differentiated the condition from progeria and mandibulofacial dysostosis [8], but that is likely to have contained a clinically heterogeneous patient group due to lack of available genetic testing. We reviewed 24 patients with HSS to review the phenotype, identify patients who could have other genetic conditions, and propose management strategies. Although defining diagnostic criteria for HSS was hampered by variable reporting of clinical details, we found congenital cataracts and/or microphthalmia, together with a characteristic facial appearance, either described as a ‘beak-like nose’ or ‘classical’ facies, or reported as a small, short or thin nose with micrognathia and/or retrognathia, to be defining features (Fig. 2). These features have been discussed by other authors ([2]; Supplementary Table 1). The presence of the characteristic eye and facial findings indicate that HSS should be considered as part of the differential diagnosis and in our view, are the most defining features of the condition, despite the relative high frequency of the other cardinal findings (Table 1). In our series, all but one patient had at least two of the four cardinal findings of proportionate short stature, hypotrichosis or sparse or thin hair, skin atrophy, and dental anomalies (Supplementary Table 1). Using these criteria, all 24 of the patients that we included would have been diagnosed clinically with HSS. Pertinent negatives still include absence of limb findings, in particular syndactyly of the 4th and 5th digits consistent with ODDD, absence of more complex eye findings, and absence of additional malformations, which may be more consistent with other diagnoses. It is also important to recognize patients with HSS within the first year of life, as later presentations may be more suggestive of progeroid conditions. This phenotype remains recognizable, although in some publications, it has diverged significantly from the original reported cases [8]. This phenomenon of ‘phenotypic divergence’ has also been noted for other conditions for which the causative gene has proven challenging to identify, for example, Dubowitz syndrome.

In a comparison with the frequency of clinical findings described by other authors, our review yielded similar results to that of Preudhomme et al. ([10]; Table 1). Although congenital cataracts were found in 20/24 (83%) of patients in this review compared to 69% in the paper of Preudhomme et al., [10], the other cardinal features of HSS were all more common in the former paper. However, no statistical comparison was possible, as Preudhomme et al. [10] did not publish the number of cases included for each clinical finding.

We identified several patients with phenotypic overlap with other conditions. The most frequent clinical overlap was with ODDD syndrome, which shares eye findings of microphthalmia and microcornea, similar facial features, and hypodontia with HSS ([24–29]). Genetic testing can be utilized to search for pathogenic variants in *GJA1* in patients with syndactyly and clinical overlap. However, sequencing of the *GJA1* gene in more than 20 patients with classic HSS did not identify any causative variants in *GJA1* and exome sequencing in the same patient group also did not identify pathogenic variants in genes associated with overlapping conditions, such as Hutchinson-Gilford progeria syndrome, Wiedemann–Rautenstrauch syndrome, mandibulofacial dysostosis, and Seckel syndrome (*LMNA*, *ZMPSTE24*, *POLR3A*, *SLC25A24*, *CEP152*, and *TCOF1* genes; [2]). We also found a cluster of patients with skeletal findings comprising natal teeth, thin and gracile tubular bones with fractures, and noted widening of the sutures with thin calvaria, limb asymmetry, and hip dysplasia in others (Supplementary Table 1). However, the number of patients was small and the findings in this group were not classical for HSS, thus it remains unclear if this group are distinct from the other reported patients.

Management guidelines for HSS have not yet been published, although potential complications related to the narrow upper airway and early pulmonary infections, respiratory compromise, OSA, and risks with anesthesia have been noted [9]. These concerns were also present in the patients that we reviewed, specifically, the prevalence of recurrent respiratory tract infections and airway diseases in 29% of the patients highlights the need for monitoring and early intervention and should inform clinical care. We have provided suggestions for management according to the commonest clinical findings (Table 3).

**Table 3:** Suggested evaluations following initial diagnosis for patients with Hallermann-Streiff syndrome

Body System	Clinical Management Considerations
Ophthalmology	• Ophthalmological examination at diagnosis. • Eye findings are managed as for other conditions • Consultation with a retinal specialist if retinal findings are present
Ear, Nose and Throat (ENT)	• ENT evaluation to assess and address potential airway and respiratory compromise • Sleep study to diagnose and monitor sleep-related concerns, such as obstructive sleep apnea • Care with anesthesia, as endotracheal intubation may be challenging
Dentistry	• Dental and orthodontist evaluation for hypodontia and other anomalies, including short dental roots • Clinical exam incl hard tissue exams & radiographs (bitewings, individualized periapicals, & orthopantomographs) can be considered • Preventive care is very important, as there may be a predisposition to severe caries • Consider referral to feeding specialist if there are clinical concerns based on severity of tooth agenesis • A craniofacial team evaluation can be considered • Imaging with computerized tomography (CT)/magnetic resonance imaging (MRI) to determine facial bone configuration
Endocrine/Growth	• Regular monitoring of growth parameters, in particular, height • Monitoring for asymmetry and scoliosis • Consultation with an endocrinologist for short stature. The efficacy of growth hormone is unknown
Dermatology	• Dermatology evaluation for management of hypotrichosis and sparse hair
Other	• Referral to immunology or to an infectious disease specialist for recurrent or severe infections • Pulmonology referral for patients with respiratory compromise

Lastly, few of the patients that we reviewed from the last 20 years were reported to have had comprehensive genetic testing and the mode of inheritance and causative gene(s) remain to be defined. HSS is still considered likely to be a genetic condition due to the high percentage of patients with bilateral congenital cataracts and the lack of any suspected environmental etiology. Pedigrees with autosomal dominant inheritance of a phenotype described as HSS have been reported (reviewed in [39]), but not all family members have had the classical facial gestalt. A female with HSS had a mother, sister, and son with cataracts but without any other anomalies [40] and several females with HSS have also had children without transmission of the clinical findings [1]. Alternatively, support for autosomal recessive inheritance in HSS has been noted, with an increased incidence of parental consanguinity [8]. There are few case reports of twins, and a pair of dizygotic twins was discordant for the HSS phenotype [41]. Non-recurrent variants in several genes in addition to *GJA1* have also been associated with an HSS phenotype. Hutchinson-Gilford progeria syndrome (HGPS) and mandibuloacral dysplasia (MAD) demonstrate phenotypic overlap with HSS [11] and are caused by de novo, missense variants in the *LMNA* and *ZMPSTE24* genes. A four-year old female diagnosed with HSS due to short stature, thin and translucent skin, macrocephaly, small hands, and camptodactyly, but no eye defects, was mosaic for c.1077dupT p.(Leu362fsX18) in *ZMPSTE24* (NM_005857.4), with 83% of exome reads showing this allele as a result of maternal uniparental isodisomy for chromosome 1 [42]. Sequencing of the genes *LMNA*, *ZMPSTE24* and *ICMT* in 8 patients with HSS revealed a single, heterozygous missense variant, c.1930 C > T predicting p.(Arg644Cys) in *LMNA* in one patient [11]. However, this allele was found in 3,247/1,613,372 controls and 13 homozygotes in gnomAD [43] and there were no suggestive findings on skin biopsy, thus pathogenicity associated with the variant seems unlikely [11]. A de novo, missense variant in *CHD*6, p.Ile1600Met, has also been identified in a patient who reportedly presented with HSS [44], but the clinical features of this individual were not provided and further pathogenic variants in this gene in HSS patients have not been described. Lastly, a novel biallelic, frameshift variant, c.2629delC p.Gln877fs was reported in *TRPM*7 in a 10-year 6-month male born to consanguineous parents with bilateral congenital cataracts, mild microcephaly with short stature, frontal bossing, deep-set eyes, a prominent, thin, pinched and tapering nose, anomalous ears, hypoplastic mandible, hypodontia, and persistent deciduous teeth [45]. There was no skin atrophy and he had severe intellectual disability with an IQ that was assessed to be 18 [45]. A sister had absent speech, cataract, and hypodontia and was homozygous for the same variant [45]. However, the lack of skin findings, anomalous ears and severe delays are not typical in HSS and the gene has been linked to neurocognitive disorders and renal disease with seizures and hypomagnesemia [46]. The high numbers of sporadic patients with HSS could also be consistent with a *de novo* germline etiology or mosaic cause for the disorder [1, 2, 10]. It is also plausible that genetic heterogeneity, epigenetics and gene regulation, gene–environment interactions, and other non-Mendelian factors contribute to the etiology [1].

## Conclusion

Our review highlights the diagnostic challenges associated with HSS due to overlap with other genetic conditions, phenotypic variability, and the relatively low number of reported cases. We found that all 24 patients studied had congenital cataracts and/or microphthalmia along with a characteristic facial appearance comprising a ‘beak-like’ nose or classical facies, often described as a small, short, or thin nose with micrognathia and/or retrognathia. Additionally, the diagnosis is supported by at least two of the following four findings: proportionate short stature, hypotrichosis or sparse/thin hair, skin atrophy, and dental anomalies. Digital anomalies, complex eye malformations and additional birth defects may be suggestive of an alternative diagnosis. However, although we could define the commonest features, it is likely that early reports of HSS were heterogenous, and it remains unclear if a single etiology will be found. Lastly, we have proposed management guidelines to address the specific needs of patients with HSS and ensure comprehensive and appropriate care.

## Supplementary Information

Below is the link to the electronic supplementary material.

Supplementary Material 1

## Data Availability

The data have been uploaded as Supplementary Material.
